# Increased risk of ischemic stroke in cervical cancer patients: a nationwide population-based study

**DOI:** 10.1186/1748-717X-8-41

**Published:** 2013-02-28

**Authors:** Shiang-Jiun Tsai, Yung-Sung Huang, Chien-Hsueh Tung, Ching-Chih Lee, Moon-Sing Lee, Wen-Yen Chiou, Hon-Yi Lin, Feng-Chun Hsu, Chih-Hsin Tsai, Yu-Chieh Su, Shih-Kai Hung

**Affiliations:** 1 Department of Radiation Oncology, Buddhist Dalin Tzu Chi General Hospital, 2, Ming Sheng Road, Dalin, Chiayi, Taiwan; 2 Department of Neurology, Buddhist Dalin Tzu Chi General Hospital, Chiayi, Taiwan; 3 Department of Allergy, Immunology and Rheumatology, Buddhist Dalin Tzu Chi General Hospital, Chiayi, Taiwan; 4 Department of Otolaryngology, Buddhist Dalin Tzu Chi General Hospital, Chiayi, Taiwan; 5 Department of Industrial Engineering and Management, National Yunlin University of Science & Technology, Yunlin, Taiwan; 6 Department of Hematology Oncology, Buddhist Dalin Tzu Chi General Hospital, Chiayi, Taiwan; 7 School of Medicine, Tzu Chi University, Hualien, Taiwan

**Keywords:** Cervical cancer, Radiotherapy, Stroke

## Abstract

**Background:**

Increased risk of ischemic stroke has been validated for several cancers, but limited study evaluated this risk in cervical cancer patients. Our study aimed to evaluate the risk of ischemic stroke in cervical cancer patients.

**Methods:**

The study analyzed data from the 2003 to 2008 National Health Insurance Research Database (NHIRD) provided by the National Health Research Institutes in Taiwan. Totally, 893 cervical cancer patients after radiotherapy and 1786 appendectomy patients were eligible. The Kaplan-Meier method and the Cox proportional hazards model were used to assess the risk of ischemic stroke.

**Results:**

The 5-year cumulative risk of ischemic stroke was significantly higher for the cervical cancer group than for the control group (7.8% *vs* 5.1%; *p* <0.005). The risk of stroke was higher in younger (age <51 years) than in older (age ≥51 years) cervical cancer patients (HR = 2.73, *p* = 0.04; HR = 1.37, *p* = 0.07) and in patients with more than two comorbid risk factors (5 years cumulative stroke rate of two comorbidities: 15% compared to no comorbidities: 4%).

**Conclusions:**

These study demonstrated cervical cancer patients had a higher risk of ischemic stroke than the general population, especially in younger patients. Strategies to reduce this risk should be assessed.

## Introduction

Cervical cancer remains an important prevalent malignant disease in women with age-adjusted incidence of 26.2 per one hundred thousand people in Taiwan. The three main methods of treatment are surgery, radiotherapy, and chemotherapy. Radiotherapy has a critical role in the primary management of patients with cervical cancer. However, definitive treatment ultimately fails in approximately 30% of cervical cancer patients [1]. Concurrent chemoradiotherapy is the standard treatment for patients with advanced cervical cancer which has better survival than radiotherapy alone or chemotherapy alone [2]. Although the number of long-term survivors has risen and continues to raise, data on the late toxicity of treatment remains limited.

Radiation-induced vascular disease had been reported. Jacobson et al. found a significantly increased incidence of thromboembolism in patients with cervical cancer [3]. In addition, Maduro et al. showed an increased risk for developing myocardial infarction [4]. Furthermore, pelvic radiotherapy for cervical cancer affects menopause [5]. These results demonstrate that radiotherapy-induced late complications can be not only local and but also systemic. Previous studies have shown that the risk of ischemic stroke is increased post-radiotherapy in breast and head and neck cancer patients [6]. However, limited study evaluated this risk in cervical cancer patients after radiotherapy. Thus, the aim of this study was to evaluate the risk of ischemic stroke in cervical cancer patients during a 5-year follow-up after radiotherapy.

## Materials and methods

### Ethics statement

The study protocol was approved by the Buddhist Dalin Tzu Chi General Hospital Institutional Review Boards. Informed consent was not needed and waived because these data analyzed consist of deidentified secondary data released to the public for research.

### Database

The study analyzed data from the 2003 to 2008 National Health Insurance Research Database (NHIRD) provided by the National Research Institutes in Taiwan. The NHIRD contains the medical benefit claims for 97% of the population and a registry of board-certified physicians and contracted medical facilities.

There were third cohorts. The principal diagnosis in the first cohort was cervical cancer identified by the International Classification of Disease, Ninth Revision, Clinical Modification (ICD-9-CM) code 180 and after radiotherapy by ICD-9-CM codes 9223, 9224. In the second cohort, who served as a control group, it was appendectomy (ICD-OP code 47). Appendectomy patients were selected as a control group here which has been adopted as control cohort in other database studies to presenting general population because of similarity to general population [7]. In addition, we also added the third cohort for comparison. The third cohort was surgery alone with oophorectomy (ICD-OP code 655, 656).

Data on each patient were collected starting from the first hospitalization or outpatient visit in 2003. Totally, 893 cervical cancer patients after radiotherapy, 1786 appendectomy patients and 379 surgery alone cervical cancer patients were eligible. Because there was a significant between-group difference in age, a group of 1786 age-matched controls was randomly selected from a pool of 10,346 appendectomy patients and stratified by age into four groups: younger than 45, 45–64, 65–74, and older than 75.

### Measurements

The primary dependent variable was ischemic stroke (ICD-9-CM codes 433–438). Other vascular events, including venous thromboembolism (VTE; ICD-9-CM codes 4538–4539), angina pectoris (AP, ICD-9-CM code 413), and myocardial infarction (MI; ICD-9-CM code 414) were also evaluated. Patients were excluded if they had vascular events before being diagnosed with cancer or had appendectomy. The date that we calculated risk was from the diagnosis of cervical cancer. Radiotherapy would be started within 4 weeks. Deaths recorded in the database were marked so as to calculate the vascular event-free survival, with cases censored if the patients died from non-vascular causes during the follow–up period. The independent variables were age, comorbidities, geographic region, urbanization level, and socioeconomic status. Comorbidities included hypertension, diabetes, coronary heart disease, and hyperlipidemia. There were four geographic regions (northern, central, southern and eastern) and three urbanization levels (urban, suburban and rural). This study also used enrollee category (EC) which was registered in NHIRD as a proxy measure of socioeconomic status. All patients were categorized as high socioeconomic status (civil servants, full-time, or regular paid personnel with a government affiliation, employees of privately owned institutions), middle (self-employed individuals, other employees, and members of the farmers’ or fishermen’s associations), and low socioeconomic status (veterans, members of low-income families, and substitute service draftees) [8]. These variables have been associated with vascular disease [9].

### Statistical analysis

The statistical software packages SAS (version 9.2; SAS Institute, Inc., Cary, NC, USA) and SPSS (version 17; SPSS Inc., Chicago, IL, USA) were used for data analysis. Between-cohort differences in frequencies of variables were evaluated using the chi-square test. Cox regression model analysis was used to calculate the effects of VTE, AP, MI, and ischemic stroke events on the experimental and control groups after adjusting for confounders. The confounders included age, comorbidities, geographic region, urbanization level, and socioeconomic status. The vascular event-free survival was calculated using the Kaplan-Meier method. *P* < 0.05 was defined as statistically significant.

## Results

The distribution of demographic characteristics and comorbidities for the cervical cancer patients after radiotherapy and appendectomy cohorts is shown in Table 1. Compared to the control group (after matching), the experimental group had a significantly lower prevalence of hypertension and coronary heart disease, but a similar prevalence of hyperlipidemia and diabetes. A higher percentage of the cervical cancer group had lower socioeconomic status and resided in suburban areas or central Taiwan. There was no significant difference in the prevalence of vascular events between the radiotherapy alone (RT; n = 382), surgery plus radiotherapy (SRT; n = 170), chemotherapy and radiotherapy (CRT; n = 268), and surgery plus chemotherapy and radiotherapy (SCRT; n = 73) groups. We also compared these patients with (CRT + SCRT) or without (RT + SRT) chemotherapy. However, there was no significant difference.

**Table 1:** Demographic characteristics and comorbidities of the cervical cancer after radiotherapy and control groups

**Variable**	**Cervical cancer after radiotherapy group**	**Control group**	***p***
	**(N = 893)**	**(N = 1786)**	
	**No. (%)**	**No. (%)**	
Age, year			NA
≦44	163 (18.3)	326 (18.3)	
45–54	230 (25.8)	460 (25.8)	
55–64	159 (17.8)	318 (17.8)	
65–74	196 (22.0)	392 (22.0)	
≧75	145 (16.2)	290 (16.2)	
Hypertension			0.01
Yes	88 (9.9)	237 (13.3)	
No	805 (90.1)	1549 (86.7)	
Diabetes			0.18
Yes	63 (7.1)	153 (8.6)	
No	830 (92.9)	1633 (91.4)	
Coronary heart disease			0.04
Yes	13 (1.5)	48 (2.7)	
No	880 (98.5)	1738 (97.3)	
Hyperlipidemia			0.68
Yes	8 (0.9)	19 (1.1)	
No	885 (99.1)	1767 (98.9)	
Geographic region			<0.001
Northern	339 (38.0)	805 (45.1)	
Central	286 (32.0)	439 (24.6)	
Southern	237 (26.5)	478 (26.8)	
Eastern	31 (3.5)	64 (3.6)	
Urbanization level			<0.001
Urban	192 (21.5)	521 (29.2)	
Suburban	421 (47.1)	733 (41.0)	
Rural	280 (31.4)	532 (29.8)	
Socioeconomic status			<0.001
High	287 (32.1)	690 (38.6)	
Middle	424 (47.5)	832 (46.6)	
Low	182 (20.4)	264(14.8)	

**Figure 1:**
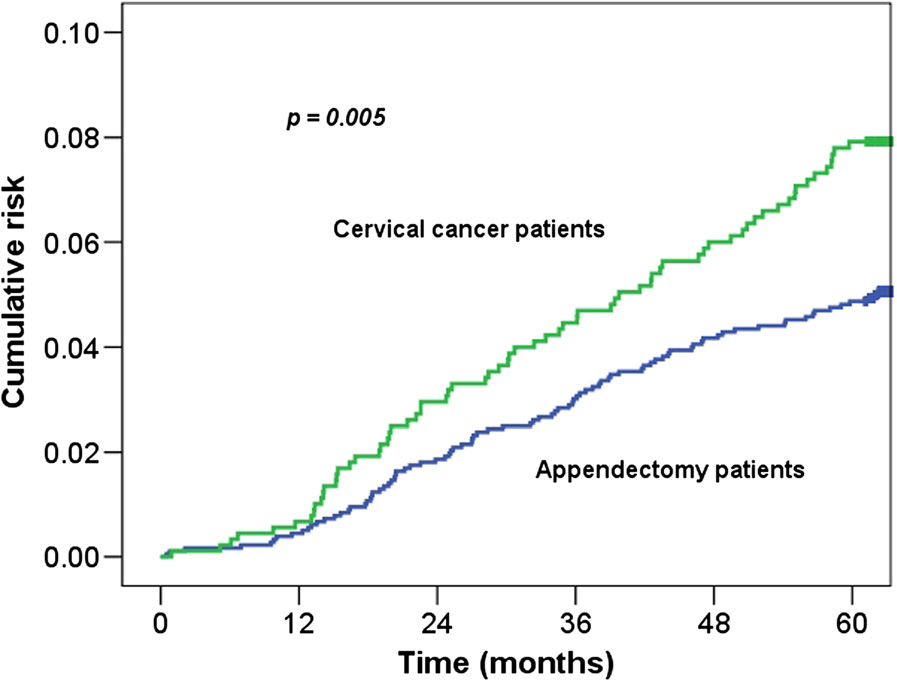
Cumulative risk of ischemic stroke in patients with cervical cancer after radiotherapy and control patients.

At the end of follow up in 2008, a total of 161 patients had ischemic strokes, including 70 (7.8%) in the cervical cancer after radiotherapy group and 91 (5.1%) in the control group. The median interval between radiotherapy and the ischemic stroke event was 32.3 months. The 5-year cumulative risk of ischemic stroke was significantly higher for the cervical cancer group than for the controls (7.8% *vs* 5.1%, *p* = 0.005; Figure 1).

Unadjusted and adjusted hazard ratio for the association of VTE, AP, MI, and ischemic stroke with experimental cohort and control cohort are shown in Table 2. After adjustments for age, comorbidities, geographic region, urbanization level, and socioeconomic status, the hazard ratio for VTE, AP, MI, and ischemic stroke during the 5-year follow-up after radiotherapy was 9.63, 3.20, 1.58 and 1.52-times higher than in the controls, respectively. We then further stratified patient in two age groups, a ≥51 years age group and <51 years age group. The risks of AP, MI, and ischemic stroke were higher in the younger cancer group than in the age-matched control group (Table 3). We then further compared the difference between surgery alone with oophorectomy and cervical cancer patients who received radiotherapy as a part of their treatment. However, there was no significant difference between them (Table 4).

**Table 2:** Crude and adjusted hazard ratios for different vascular events in the 5-year follow-up period

		**Events**	**Unadjusted**	***p***	**Adjusted**	***p***
		**(%)**	**HR (95%CI)**		**HR (95%CI)**	
VTE	Control group (N = 1786)	6 (0.34)	1		1	
	Cervix Cancer after radiotherapy group (N = 893)	30 (3.4)	10.17 (4.24–24.4)	<0.001	9.63 (3.98–23.3)	<0.001
AP	Control group (N = 1786)	22 (1.2)	1		1	
	Cervix Cancer after radiotherapy group (N = 893)	31 (3.5)	2.85 (1.65–4.92)	<0.001	3.20 (1.83–5.60)	<0.001
MI	Control group (N = 1786)	85 (4.8)	1		1	
	Cervix Cancer after radiotherapy group (N = 893)	66 (7.4)	1.58 (1.14–2.17)	0.006	1.58 (1.14–2.20)	0.006
Ischemic stroke	Control group (N = 1786)	91 (5.1)	1		1	
	Cervix Cancer after radiotherapy group (N = 893)	70 (7.8)	1.56 (1.14–2.13)	0.005	1.52 (1.10–2.08)	0.01

Adjusted for age, hypertension, diabetes, coronary heart disease, hyperlipidemia, geographic region, urbanization level, and enrollee category.

VTE indicates venous thromboembolism; AP indicates angina pectoris, and MI indicates myocardial infarction.

**Table 3:** Adjusted hazard ratios for different vascular events compared between age matched-controlled groups

	**<51 years old**		**≥51 years old**	
	**Adjusted HR (95%CI)**	***p***	**Adjusted HR (95%CI)**	***p***
Control Groups	1		1	
AP	4.24 (1.03–17.54)	0.04	3.09 (1.67–5.71)	<0.001
MI	4.59 (1.74–12.10)	0.002	1.35 (0.95–1.94)	0.09
Ischemic stroke	2.73 (1.04–7.13)	0.04	1.37 (0.97–1.93)	0.07

Adjusted for age, hypertension, diabetes, coronary heart disease, hyperlipidemia, geographic region, urbanization level, and enrollee category.

VTE indicates venous thromboembolism.

**Table 4:** Crude and adjusted hazard ratios for different vascular events in the 5-year follow-up period

		**Events**	**Unadjusted**	***p***	**Adjusted**	***p***
		**(%)**	**HR (95%CI)**		**HR (95%CI)**	
VTE	Surgery alone with oophorectomy group (N = 379)	15 (4.0)	1		1	
	Cervix Cancer after radiotherapy group (N = 893)	30 (3.4)	1.06 (0.57–1.98)	0.84	1.05 (0.56–1.97)	0.87
AP	Surgery alone with oophorectomy group (N = 379)	19 (5.0)	1		1	
	Cervix Cancer after radiotherapy group (N = 893)	31 (3.5)	0.94 (0.52–1.71)	0.94	0.83 (0.45–1.53)	0.55
MI	Surgery alone with oophorectomy group (N = 379)	44 (11.6)	1		1	
	Cervix Cancer after radiotherapy group (N = 893)	66 (7.4)	0.90 (0.60–1.34)	0.60	0.80 (0.53–1.21)	0.30
Ischemic stroke	Surgery alone with oophorectomy group (N = 379)	31 (8.2)	1		1	
	Cervix Cancer after radiotherapy group (N = 893)	70 (7.8)	1.21 (0.79–1.86)	0.36	0.97 (0.62–1.51)	0.90

Adjusted for age, hypertension, diabetes, coronary heart disease, hyperlipidemia, geographic region, urbanization level, and enrollee category.

VTE indicates venous thromboembolism; AP indicates angina pectoris, and MI indicates myocardial infarction.

**Figure 2:**
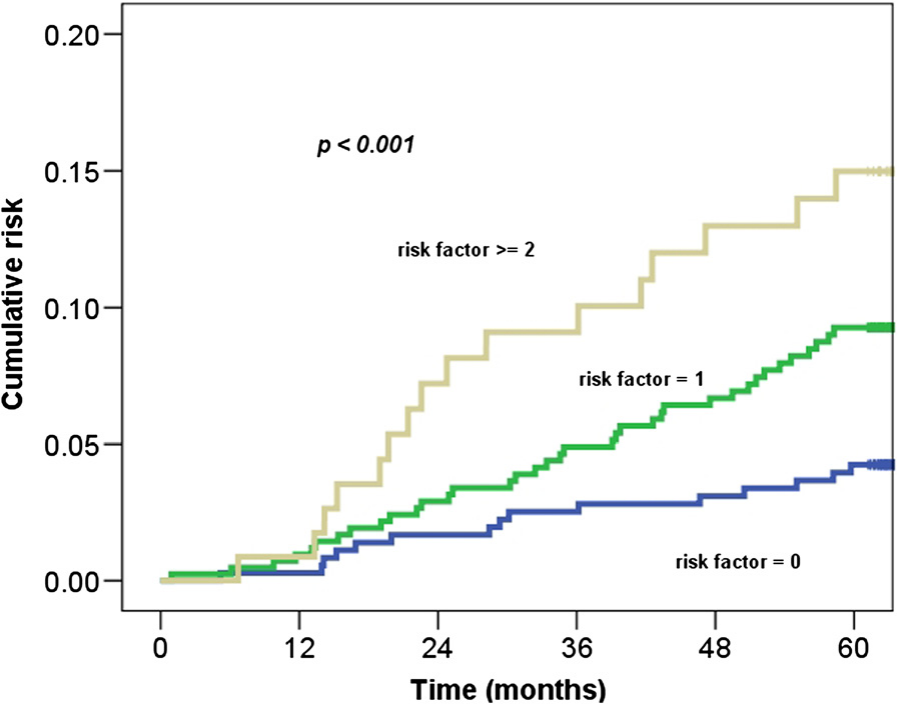
The risk of stroke in cervical cancer patients with ≧2 risk factors compared with that in patients with 0 or 1 risk factor.

Five stroke-related risk factors (age older than 55 years, hypertension, diabetes, coronary artery disease, and hyperlipidemia) were further used to stratify the cervical cancer cohort into 3 groups: a low-risk group (n = 360; no risk factor), intermediate-risk group (n = 418; 1 risk factor), and high-risk group (n = 115; ≥2 risk factors). The 5-year cumulative risks of stroke in the stratified groups were 4%, 9%, and 15%, respectively. Figure 2 shows that the risk of stroke was higher for cervical cancer patients with ≧2 risk factors than for patients with 0 or 1 risk factor (*p* < 0.001).

## Discussion

Cervical cancer is an important health problem. Although its incidence is decreasing, it remains the leading cause of death from cancer in women in Taiwan. Radiotherapy is an integral component of the standard treatment, particularly treatment of bulky tumors and advanced stage disease. The absolute survival benefit of platinum-based concurrent chemoradiation in locally advanced cervical cancer has been reported to be 12% [2]. Although radiotherapy and chemotherapy could increase tumor control, they also increase local and systemic toxicity. Several investigators have mentioned the importance of recording therapy-related toxicity. However, the data are limited, particularly the data on late effects [10]. Our study demonstrated that cervical cancer patients who received radiation therapy as part of their cancer treatments had a higher risk of VTE, AP, MI, and ischemic stroke compared with the general population.

Associations of stroke risk with treatment modalities in different cancers have recently been reported. Nilsson et al. reported a statistically significant increase in the risk of stroke in women with a history of breast cancer [6]. Dorresteijn et al. showed that radiation to the neck was associated with a 5.6 times increased risk of ischemic stroke after a median follow-up time of 7.8 years [11]. Julio et al. reported the later development of disseminated intravascular coagulation-mediated cerebral infarction in a patient with cervical cancer [12]. However, there have been no population-based reports regarding the correlation of stroke and cervical cancer. This is the first large study to estimate the incidence of stroke in cervical cancer patients treated with radiotherapy.

Radiotherapy in cervical cancer patients not only has local but also systemic late effects. Vascular damages induced by radiation have been much investigated. Radiation could induce vascular damage directly and also result in various types of functional damage. The effects include degeneration of endothelium, decrease in intimal thickness, splitting of the basement membrane, lipid deposits, adventitial fibrosis, and occlusion [13]. In addition, ovarian is very radiation-sensitive organ. Ovarian insufficiency caused by pelvic radiation has been well known [14]. Ovarian insufficiency has a large effect on the health of women, in particular effects on bone density, and on cardiovascular and neurological systems [15]. Furthermore, menopause, a manifestation of ovarian insufficiency, has been reported as a risk factor for stroke because of its potential for increasing blood pressure, obesity, insulin resistance, and accelerated changes of lipids and lipoproteins [16,17]. All of these effects may lead to the development of vascular events. In this cohort study, increased risks for developing AP, MI, and ischemic stroke were observed. The mean age of women at menopause in our country is 50 to 51 years old [18]. To define clearly clarify the systemic influence of menopause, patients were divided into ≥51 years and <51 years age groups. Interestingly, younger patients had 1.4, 3.4, and 2 times the risk of AP, MI, and ischemic stroke events, respectively, compared with older patients. In addition, there were no significantly different vascular events between surgery alone with oophorectomy and cervical cancer patients who received radiotherapy as a part of their treatment. These results suppose that radiotherapy in cervical cancer not only has local but also systemic effects, especially in younger patients. We supposed that ovarian insufficiency plays an important role in the development of these vascular diseases, especially in ischemic stroke. Recently, Gross et al. reported the surgical technique of ovarian transposition (moving the ovaries away from the field of irradiation) minimizes the radiation dose and damage to the ovary [19]. In addition, although the “timing hypothesis” holds that estrogens have beneficial effects on young and healthy blood vessels of women, direct evidence showing the benefit of replacement therapy is scarce [20]. Hormone replacement therapy or transposition of ovaries before radiotherapy should be considered as part of a multidisciplinary approach, especially in younger patients. However, these results and suggestions need further investigation.

Compared with the general population, cancer patients are often observed to have lower socioeconomic status [21]. This has subsequently been associated with a higher prevalence of comorbidities, such as diabetes mellitus, hypertension, or hyperlipidemia. These factors exacerbate vascular disease. In our study, five stroke-related risk factors were used to stratify the cancer patients into three groups (low-, intermediate-, and high-risk groups). The 5-year stroke incidence was lower in the low risk group, 4%, than in the intermediate risk, 9%, and high risk groups, 15%. Patients with more comorbidity had higher risk of stroke. Therefore, interventions aimed at stroke prevention are extremely important. Complete survey of modifiable risk factors and intensive lifestyle modification are indicated in patients with multiple comorbidities. Further studies are recommended to determine the role of medications used in primary prevention of ischemic stroke.

Several limitations of this study should be mentioned. First, hospitalized or outpatients with a principal diagnosis of cervical cancer were chosen to avoid inclusion of patients with misdiagnosed cervical cancer, though some patients may have been missed. Second, in our multivariate analysis, increase in the incidence of stroke or any other vascular events was unrelated to the addition of platinum-based chemotherapy to radiotherapy. The relatively small size of the census populations and the relatively short follow-up period probably hindered the analysis, but we have found the significant different stroke rate in these two cohorts in this short period. Third, the NHIRD has no data on clinical characteristics, including staging, stroke severity, and biochemical data or other information, like tobacco use, dietary habits, body mass index, and activity level (ECOG) for further analysis. Smoking is the important factor for cervical cancer and vascular events. Tsai et al. reported the prevalence of smoking increased gradually from 3% in the normal group, 9% in the inflammation group and 13.6% in the intraepithelial neoplasm group in Taiwan [22]. Strategies to reduce these risks should be considered, especially in younger patients. Although we had many limitations; however, these would not change our conclusion, given the magnitude and statistical significance of the observed effects in this study.

## Conclusion

In this cohort study, the risk of VTE, AP, MI, and ischemic stroke was significantly higher in cervical cancer patients who received radiation therapy as part of their cancer treatments, especially in younger patients. Strategies to reduce these risks need to be further examined.

## Competing interests

The authors made no disclosures.

## Authors’ contributions

TSJ, HYS, TCH and HSK developed the ideas for these studies, performed much of the work, and drafted the manuscript. LCC, LMS and HSK revised the manuscript. CWY, LHY and SYC designed the study, managed and interpreted the data. TSJ, TCH and HFC performed the statistical analysis. All authors read and approved the final manuscript.
